# Efficacy of a Web-Based Psychoeducational Intervention for Young Adults With Fertility-Related Distress Following Cancer (Fex-Can): Randomized Controlled Trial

**DOI:** 10.2196/33239

**Published:** 2022-03-29

**Authors:** Claire Micaux, Maria Wiklander, Lars E Eriksson, Lena Wettergren, Claudia Lampic

**Affiliations:** 1 Department of Women’s and Children’s Health, Karolinska Institutet Stockholm Sweden; 2 Department of Neurobiology, Care Sciences and Society Karolinska institutet Stockholm Sweden; 3 Department of Learning, Informatics, Management and Ethics, Karolinska Institutet Stockholm Sweden; 4 Medical Unit Infectious Diseases, Karolinska University Hospital Huddinge Sweden; 5 School of Health Sciences, City University of London London United Kingdom; 6 Department of Public Health and Caring Sciences, Uppsala University Uppsala Sweden; 7 Department of Psychology, Umeå University Umeå Sweden

**Keywords:** cancer, fertility distress, psychoeducation, randomized controlled trial, web-based

## Abstract

**Background:**

Threatened fertility following cancer diagnosis in the reproductive age may severely impact emotional and psychosocial well-being in survivorship. Effective web-based interventions for fertility-related distress have been lacking.

**Objective:**

This study aims to test whether the Fertility and Sexuality following Cancer (Fex-Can) intervention is superior to standard care in reducing fertility-related distress and related psychosocial outcomes in young adults with cancer.

**Methods:**

This randomized controlled trial evaluated a 12-week, web-based, automated self-help intervention for fertility-related distress following cancer—Fex-Can Fertility. Individuals were identified via Swedish national quality registries, and those reporting fertility-related distress 1.5 years after diagnosis were invited. A total of 100 women and 24 men (aged 19-40 years) answered self-administered surveys at baseline (T0), directly after the intervention (T1), and 3 months later (T2). The main outcome was fertility-related distress, which was measured by using the 6-dimension Reproductive Concerns After Cancer (RCAC) scale. The secondary outcomes were health-related quality of life (European Organization for Research and Treatment of Cancer Quality of Life Questionnaire), emotional distress (Hospital Anxiety and Depression Scale), fertility-related knowledge, and fertility self-efficacy. In addition, the intervention group (IG) reported self-perceived changes in problems related to fertility after cancer (T1). 2-tailed *t* tests and linear mixed models, including intention-to-treat and subgroup analyses, were performed to compare the effects of the intervention with those of standard care.

**Results:**

Although 62% (31/50) of the participants in the IG stated that their concerns about fertility were fewer after the intervention, there were few statistically significant group differences in the main outcome (RCAC) at T1 and T2. Compared with controls, the IG rated lower distress concerning the dimension child’s health at T2 (*P*=.003; effect size [ES]=0.64). This difference was maintained when adding group and time interactions (intention-to-treat: *P*=.003; ES=0.58). The IG also had better self-perceived cancer-related fertility knowledge at T1 (*P*=.05; ES=0.35) and T2 (*P*=.01; ES=0.42) than the control group. Subgroup analyses based on dose or adherence and baseline RCAC scores did not substantially alter these results. Overall, the use of the web-based program was low.

**Conclusions:**

The Fex-Can intervention had small to moderate positive effects on cancer-related fertility knowledge and distress related to child’s health. The lack of group differences in other dimensions of fertility distress and related secondary outcomes contrasted with reports on self-perceived improvement after the intervention. The Fex-Can Fertility program may be a useful complement to routine psychosocial support in the clinical care of young women and men with cancer.

**Trial Registration:**

ISRCTN Registry 36621459; https://www.isrctn.com/ISRCTN36621459

## Introduction

### Background

Physiological and psychological changes following cancer diagnosis and its treatment may have detrimental effects on reproductive health [1,2]. Concerns about fertility and parenthood are among the top unmet care needs of young people diagnosed with cancer, regardless of diagnosis and gender [3]. Reproductive concerns include topics such as uncertainty about one’s own fertility potential, concerns about how to tell a current or potential partner about impaired fertility, the fear of recurrence or of one’s own health as a barrier to taking care of a family, and distress related to the risk of transmitting cancer genetically to future children [4,5]. Concerns related to fertility and parenthood have been shown to correlate with depressive symptoms [6] and health-related quality of life [3,7-9] in young adults diagnosed with cancer, especially when there is an unfulfilled wish for a child [3,8,10]. Previous research suggests that unmet information needs regarding reproductive health constitute a central aspect negatively affecting the quality of life of women and men diagnosed with cancer in the reproductive age and contribute to fertility-related distress [7]. Intervening with reliable, relevant, and timely information and psychoeducation should, therefore, be the first step toward preventing or alleviating fertility distress. Fertility distress has been studied in qualitative and quantitative research [3,11], and self-administered questionnaires have been developed to measure the phenomenon and study its relationship with other psychosocial variables, such as depressive symptoms and health-related quality of life [4,10,12].

Psychosocial interventions for cancer survivors, which may or may not include web-based components, often have a broad scope [13] and are referred to as *survivorship care plans* [14], self-management interventions [15], or multidimensional programs [16,17]. There is a shortage of interventions targeting both medical and psychosocial concerns regarding fertility and parenthood following cancer. A systematic review of fertility-related psychological distress following cancer reported only 3 psychological interventions [3]. The only web-based intervention study in the field of fertility after cancer that we know of was limited to an educational focus and a single diagnosis (women with breast cancer). The intervention consisted of educational modules aiming to raise participants’ knowledge about reproductive health, a web-based bulletin board (discussion forum), and the possibility of interacting with researchers. The study had a noncontrolled, pre-post design and was published as early as 2010 [18].

In the past decade, eHealth has exploded as a research and clinical discipline, and the number of psychosocial and psychological interventions has increased. Several reviews have pointed out the complex nature of eHealth interventions and the challenges involved in their testing and implementation [19-21]. For example, there is limited evidence on dose and adherence measures [22]. The internet is a suitable arena for reaching people in remote areas and approaching private issues; therefore, web-based interventions seem ideal for the topic of fertility and parenthood following cancer. Despite the growing number of web-based interventions in cancer care and survivorship [19,23-26], specific and updated knowledge on the potential of treating fertility-related distress over the internet remains scarce [27].

It has been suggested that to be effective, complex eHealth interventions need to be underpinned by an explicit theoretical framework reflected in the proposed behavior change methods [13,28] and in the choice of outcomes [29]. It has been pointed out that interventions for cancer survivors often lack a theoretical framework and are heterogeneously designed, precluding a thorough evaluation of their working mechanisms [30]. To extend the evidence base, the Fertility and Sexuality following Cancer (Fex-Can) intervention was developed in a participatory process engaging former patients with cancer as research partners [31]. The intervention was conceived in line with the holistic framework for eHealth intervention development [32] and underpinned by the tenets of the self-determination theory (SDT) [33]. According to the SDT, there are 3 universal basic psychological needs—competence (feeling capable), relatedness (feeling connected to others), and autonomy (feeling able to act according to one’s inner will). To achieve sustained behavior change and general psychological well-being, all basic needs must be satisfied [29,33]. Therefore, an intervention designed to make participants feel more competent, related to others, and autonomous in relation to decisions surrounding one’s fertility was presumed to be effective in the long term. Self-efficacy is presumed to be a proxy measure for competence [34], as it includes not only confidence in knowledge but also the perceived ability to handle actual situations [35]. This confidence in one’s capability has been suggested as a mediator for making informed choices and finding motivation for sustainable behavior change in cancer survivorship [36].

The intervention went through feasibility testing [37] and was deemed suitable for the intended target population: women and men aged 19 to 41 years with a recent history of one of the following diagnoses: breast, gynecologic or testicular cancer, lymphoma, or central nervous system tumors.

### Objectives

The aim of this study is to test the efficacy of the Fex-Can intervention in reducing fertility-related distress and psychosocial outcomes in young adults with cancer.

The specific research questions are as follows:

1. Is the Fex-Can Fertility program superior to standard care in reducing fertility distress directly after the end of the program and 3 months later?
2. Does the Fex-Can Fertility program increase fertility self-efficacy and fertility-related knowledge, reduce emotional distress, or improve health-related quality of life compared with standard care?
3. Do baseline levels of fertility distress predict the effect of the program over time?
4. Does dose, that is, the uptake and adherence to the program, influence the change in fertility distress ratings over time?

## Methods

### Trial Design

The Fex-Can project encompasses a national cohort study [38] with an embedded randomized controlled trial (RCT) including participants with self-reported distress or dysfunction at baseline [39]. The Fex-Can web-based psychoeducational program was offered in two versions—Fex-Can Sex and Fex-Can Fertility, with the latter being evaluated in this study. A detailed description of the study design is available in 2 published study protocols [38,39] and is briefly described in next sections. The Fex-Can Fertility trial is reported here by combining the Template for Intervention Description and Replication checklist [40] with guidelines for eHealth interventions [41] and social and psychological interventions [42], which are both extensions of the original 2010 CONSORT (Consolidated Standards of Reporting Trials) statement for reporting randomized trials [43]. The trial was registered on January 25, 2016 (trial number: 36621459).

### Sample

The sample was drawn from a cohort of 1499 individuals diagnosed with breast, cervical, ovarian, or testicular cancer; lymphoma; or central nervous system tumor between 2016 and 2017, approximately 1.5 years before the start of the study. The time frame was chosen to approach people who were likely to have finished primary treatment but were still close enough to diagnosis to be in need of psychosocial support. Eligible participants were identified using Swedish national quality registries, and all people in the intended age bracket (18-39 years at diagnosis) were approached for a longitudinal cohort study, with a letter containing a survey sent to their population registration address. The survey could be completed either on paper or via the web and included written informed consent. Individuals reporting fertility distress at the baseline assessment were invited to the Fex-Can Fertility trial and had to send a signed form back, granting their consent to participate in the RCT.

### Eligibility

Respondents scoring ≥4 on at least 1 subscale of the Reproductive Concerns After Cancer (RCAC) scale [4] were eligible for the RCT.

### Allocation

**Figure 1:**
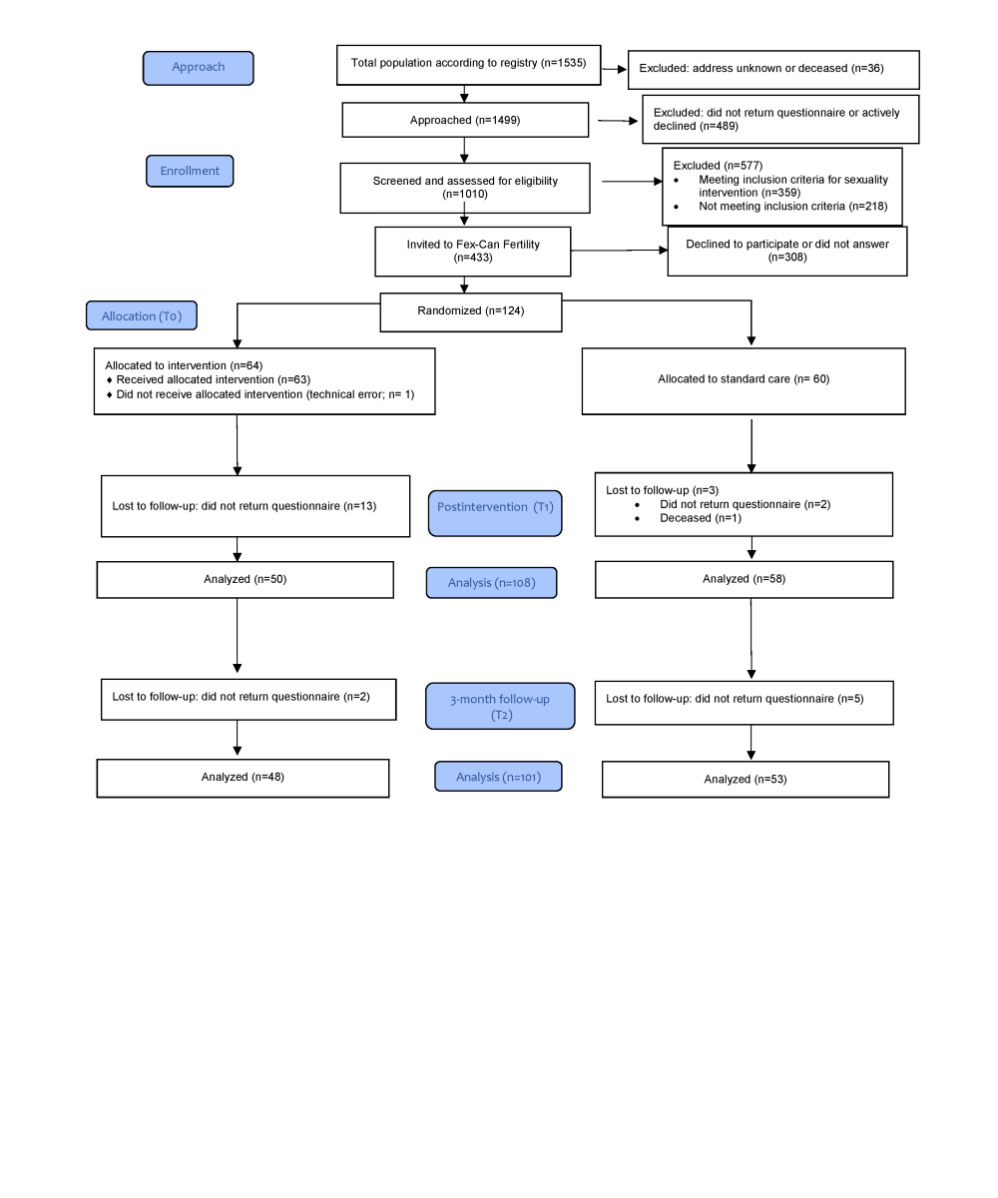
Flow of participants—CONSORT SPI-2018 (Consolidated Standards of Reporting Trials Statement for Social and Psychological Interventions) flow diagram.

Allocation (1:1 ratio) to either the intervention group (IG) or control group (CG) was performed by an external statistician uninvolved in the data collection process by stratified block randomization, taking into account sex and diagnosis. Owing to the design of the intervention, a placebo condition was not possible and neither participants nor researchers could be blinded to the group allocation. Participants were considered lost to follow-up only if they, for any reason, did not return the postintervention questionnaires; therefore, no pattern of attrition was determined after randomization. The flow of participants is summarized in Figure 1.

The sample size was estimated to be 128 individuals needed at follow-up, to obtain statistically significant results, assuming 80% power, medium effect size (ES; 0.5), and a significance level set at .05. As the attrition rate between baseline and first follow-up was expected to be around 15%, we aimed to include 210 participants at baseline.

### Intervention

The intervention was a 12-week, web-based psychoeducational program. The Fex-Can Fertility program was organized in 6 successive modules with informational material, texts, and exercises aiming at developing competence and facilitating behavior change through a sound balance between change and acceptance strategies. The modules covered known aspects of fertility distress [4] and were entitled *Fertility after cancer, Handling anxiety, Trying to have children after cancer, My own health and my child’s health, Not being able to have biological children,* and *Relationships.* Contents are described in detail in a doctoral thesis aiming for a process and outcome evaluation of the Fex-Can Fertility intervention [44]. The design, content, and mode of delivery were conceived to facilitate the satisfaction of participants’ basic needs according to the SDT [33]. It was assumed such theoretical orientation would enhance positive health outcomes such as self-efficacy and health-related quality of life [29]. Nuanced information and reliable facts were intended to leverage participants’ competence. Written and filmed survivor stories, as well as interactive quizzes and a discussion forum, were included with the goal of helping participants find strategies to handle their concerns surrounding fertility and family building after cancer by strengthening autonomy and relatedness. The development, design, content, and structure of the intervention have been described in detail in previous studies [31,37,44]. The discussion forum was moderated by one of the research partners [31] and by a member of the research team with clinical expertise in psychology or nursing. Adherence was defined using quantitative activity parameters retrieved from website system data.

### Control

The control condition was standard care, which may or may not have included fertility-related support and scheduled contacts with health care, depending on the diagnosis and treatment.

### Data Collection

#### Sociodemographic and Clinical Data

Participants were assessed on outcome measures and sociodemographic variables via a self-administered survey on the following three occasions: baseline (T0), directly after the intervention (T1), and 3 months later (T2). In addition, treatment intensity according to an adapted version of the intensity treatment rating scale [45,46] was assessed using the National Cancer Quality Registry data.

#### Main Outcome Measure (Fertility Distress)

The RCAC scale was developed for women in the United States with various cancer diagnoses [4] and has been validated for women in China [47] and Sweden [48] and for men in the United States [12]. The scale consists of a total score and six 3-item dimensions related to fertility, pregnancy, and parenthood after cancer: fertility potential (concerns about one’s ability to become a biological parent), partner disclosure (concerns related to telling a partner about possibly impaired fertility), child’s health (concerns for a biological child’s health in relation to the parent’s previous cancer diagnosis and treatment, specifically genetic risks), personal health (concerns related to fear of not being able to or living long enough to raise a child), acceptance (the extent of reconciliation with not being fertile or not having biological children), and becoming pregnant (concerns related to efforts involved in achieving a pregnancy). Responses are given on a 5-point scale ranging from strongly disagree (1) to strongly agree (5), where higher scores indicate higher levels of concern. The mean of the total score and the mean scores for each of the 6 dimensions, as recommended in a validation study of the RCAC [49], were used as primary outcomes for the Fex-Can Fertility trial.

#### Secondary Outcome Measures

##### Health-Related Quality of Life

Health-related quality of life was measured using the validated [50] summary score (range 0-100) of the European Organization for Research and Treatment of Cancer Quality of Life Questionnaire (version 3.0), which is a generic instrument developed for clinical trials regardless of cancer type [51].

##### Emotional Distress

The Hospital Anxiety and Depression Scale is a widely used scale measuring anxiety (7 items) and depression (7 items), validated for use in patients with cancer [52]. Scores are given on a numbered Likert scale ranging from 0 to 3, and for each subscale, a total score of 0 to 21 (with higher values indicating more anxiety or depressive symptoms) is calculated.

##### Fertility Self-efficacy

Perceived confidence in one’s ability to manage situations and emotions related to the threat of infertility was measured using a study-specific questionnaire based on previous research [53,54], including 6 items with statements such as “I feel confident that I can tell other people I’m concerned about my reproductive ability.” All the items are available in Multimedia Appendix 1. Answers were given on a 4-point Likert scale, with alternatives ranging from completely disagree (1) to completely agree (4). Exploratory factor analysis (data not shown) indicated that one of the items was poorly correlated with the others. The mean score was calculated for the 5 remaining items, with higher values indicating higher levels of fertility-related self-efficacy.

##### Fertility-Related Knowledge

The perceived level of knowledge concerning fertility issues was measured using a study-specific questionnaire developed from previous research [18], which consisted of 10 items. Answers were given on a 4-point Likert scale, with alternatives ranging from completely disagree (1) to completely agree (4). Exploratory factor analysis (data not shown) of the total cohort of eligible participants indicated that it was suitable to divide the scale into two domains: one for general fertility-related knowledge (4 items) and the other for cancer-related fertility knowledge (6 items). Items included statements such as “I have good knowledge regarding the menstrual cycle and when a pregnancy can occur” (general fertility knowledge) and “I have good knowledge regarding the effect of cancer and cancer treatments on reproductive ability” (cancer-related fertility knowledge). All the items are available in Multimedia Appendix 1. The means were calculated for each subscale, with higher mean scores indicating better perceived knowledge.

#### Postintervention Evaluation Survey

At T1, participants who had been randomized to the IG were presented study-specific items concerning their experience of the program. Specifically, they were asked to rate their own perceptions of how their problems regarding having children after cancer had changed compared with before participating in the program. Answers were given on a 7-point Likert scale (*Improved a lot, Improved, Improved a little, Did not change, Worsened a little, Worsened,* or *Worsened a lot*).

### Data Analysis

Data were analyzed using descriptive and inferential statistics. Statistical analyses were performed by external statisticians on blinded data. Missing data were treated as follows: for single items that were missing, we imputed according to the individual’s mean on the scale, provided half or more of the items had been answered. We chose not to impute for individuals where the entire scale was lacking (1-3 participants per group). *t* tests (2-tailed) were used to determine any significant differences between the IG and CG at baseline (T0), directly after the 12-week intervention (T1) and 3 months later (T2). A *P* value inferior or equal to .05 was considered statistically significant. Clinically important changes were calculated using Cohen *d* [55] for ESs, where the difference between the IG and CG mean scores was divided by the pooled baseline SD [56]. ESs of 0.2 to 0.5 were considered small, 0.5 to 0.8 was considered medium, and >0.8 was considered large [55].

Linear mixed models were then used to analyze possible changes over time within and among the treatment groups on the main outcome measure. Mixed models consider the potential dependence of repeated observations within participants and compensate for missing data without the need for imputation [57]. The mixed models included a participant-specific random intercept. The primary end point was T0 (baseline). All available data were used, and the analysis was based on the intention-to-treat principle. In all, 2 types of subgroup analysis were performed. First, for each dimension, participants were assigned to either *high RCAC* (≥4) or *low RCAC* (<4) on the subscale mean at baseline. In the second subgroup analysis, participants were stratified based on three levels of adherence to the program: high, low, and control. High activity was defined as having opened at least half of the modules and spent a total of at least 20 minutes on the website (general activity) plus one of the following: having spent ≥3 minutes in the discussion forum, written a post in the forum, or answered ≥50% of the quizzes (interactivity). All participants who did not meet these criteria were categorized as having *low activity*, which could also include not having logged on to the program at all. For the linear mixed models, ESs were calculated when possible by dividing the point estimate of the group difference by the residual variance. Data were analyzed using SPSS (version 26; IBM Corporation) and Stata (version 16; StataCorp LLC).

### Ethical Considerations

This study was approved by the Regional Board of Ethics in Stockholm (permit numbers: 2013/1746-31/4, 2014/224-32, and 2017/916-32) and performed in accordance with the ethical standards laid down in the 1964 Declaration of Helsinki and its later amendments.

## Results

### Participants

Eligible participants were persons aged 19 to 40 years, approximately 1.5 years after diagnosis with selected cancer types and reporting elevated levels of fertility distress in a population-based survey. Of the 433 eligible participants approached, 124 (28.6%) agreed to participate. The final sample consisted of 124 individuals, 24 (19.4%) men and 100 (80.6%) women. One participant was assessed at baseline but was excluded from follow-up due to technical failure. Participant characteristics, including sociodemographic and clinical variables, are summarized in Table 1.

Randomization resulted in 64 patients in the IG and 60 in the CG. The attrition was lower than anticipated in the power calculation. At follow-up, of 124 participants, there were 108 (87.1%) and 101 (81.5%) responses from the IG and CG at T1 and T2, respectively (Figure 1).

At baseline, there were no statistically significant differences between the IG and CG in background variables or outcome measures. Breast cancer was the most common diagnosis among participants. Most of the participants had a partner, were working as their main occupation, and had a university or college level of education. Approximately half (29/64, 45% in the IG and 35/60, 58% in the CG) of the participants already had biological children. More than half of the participants (66/124, 53.2%) had received treatments that were very or most intensive or extensive.

**Table 1:** Demographic and clinical characteristics recorded at the baseline assessment (T0; N=124).

Characteristics		Intervention group (n=64)	Control group (n=60)
**Sex, n (%)**			
	Men	13 (20)	11 (18)
	Women	51 (80)	49 (82)
Age (years), median (range)		33 (20-41)	34 (19-40)
**Country of birth, n (%)**			
	Sweden	54 (84)	51 (85)
	Another European country	3 (5)	6 (10)
	Outside Europe	7 (11)	3 (5)
**Educational level, n (%)**			
	University	39 (61)	34 (57)
	High school	20 (31)	19 (32)
	Secondary school or other	5 (8)	7 (12)
**Main occupation, n (%)**			
	Working full-time or part-time	42 (66)	44 (73)
	Student	4 (6)	4 (7)
	On sick leave	17 (27)	11 (18)
	Other (eg, unemployed or full parental leave)	1 (2)	1 (2)
**Diagnosis, n (%)**			
	Breast cancer	26 (41)	26 (43)
	Brain tumor	8 (13)	6 (10)
	Cervical cancer	10 (16)	12 (20)
	Lymphoma	11 (17)	8 (13)
	Ovarian cancer	3 (5)	2 (3)
	Testicular cancer	6 (9)	6 (10)
**Ongoing antitumoral treatment (self-reported), n (%)**			
	None	40 (63)	39 (65)
	Chemotherapy	3 (5)	2 (3)
	Radiation	1 (2)	2 (3)
	Hormonal treatment	19 (30)	17 (28)
	Other (eg, antibodies)	6 (9)	4 (7)
**Treatment intensity^a^, n (%)**			
	Level 1: least intensive or extensive treatment	10 (16)	11 (19)
	Level 2: moderately intensive or extensive	20 (33)	13 (22)
	Level 3: very intensive or extensive	29 (4)	33 (56)
	Level 4: most intensive or extensive	2 (3)	2 (3)
**Partner, n (%)**			
	Partnered	50 (78)	52 (88)
	Nonpartnered	14 (22)	7 (12)
**Parenthood status, n (%)**			
	Live with children	30 (47)	38 (63)
	Had biological children before onset of cancer	29 (45)	35 (58)
	Became a parent after cancer	3 (5)	2 (3)

^a^According to the adapted version of the intensity treatment rating scale.

### Use of the Intervention

Of the 64 participants who were randomized to the IG, 21 (33%) reached the level of use defined as *high activity*. Among the remaining 43 participants, 33 (77%) had a lower activity level and 10 (23%) had not logged on to the website at all. With regard to the activity in the discussion forum,17% (11/64) of the participants had made at least 1 posting and 31% (20/64) of the participants had been actively reading the posts for >3 minutes.

### Differences Among Groups After Intervention

#### Primary Outcome

Linear mixed models using a random intercept and based on intention to treat were conducted to study the effects of time and group on the evolution of the main outcome measure. The results are presented in Table 2.

**Figure 2:**
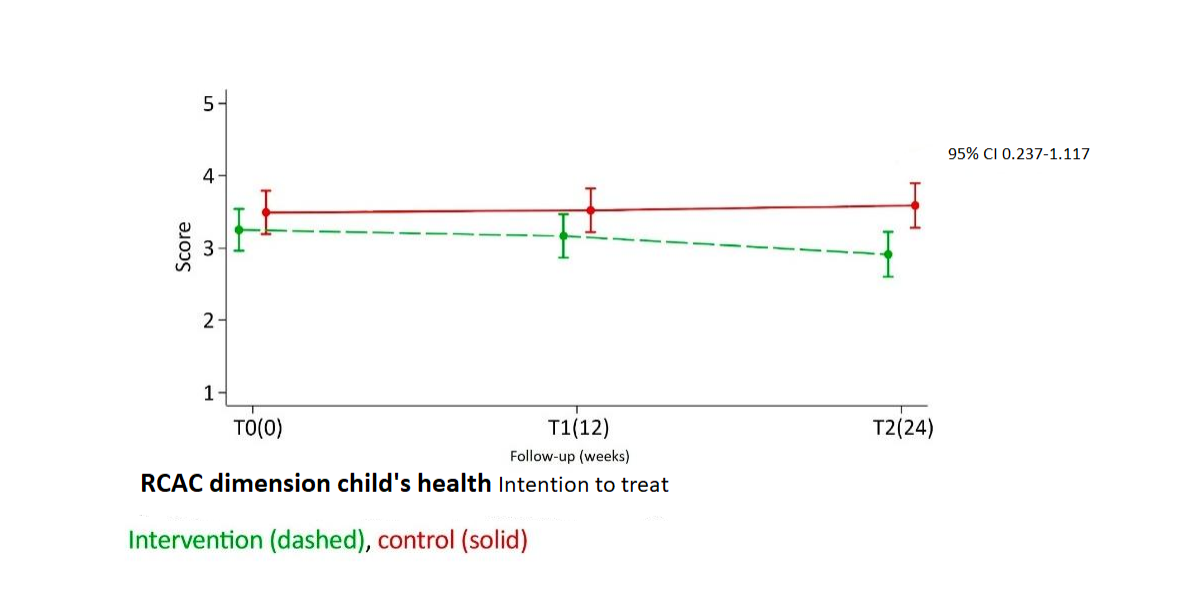
Group differences in dimension 3 (child’s health). RCAC: Reproductive Concerns After Cancer.

In intention-to-treat analyses, *child’s health* was the only dimension in which a significant group difference was detected. The IG had a decrease in scores (ie, reported fewer concerns), and the CG had a slight increase in scores (more concerns) over time (Figure 2; Table 2). At T2, the difference was significant with a moderate ES (*P*=.003; ES=0.576).

Including RCAC baseline scores and activity in the program did not substantially change the results and did not produce any clear pattern (data available in Multimedia Appendices 2-5).

**Table 2:** Difference in mean values between groups over time (linear mixed models with random intercept: group and time interaction; intention-to-treat: intervention group [IG] vs control group [CG]).

Outcome measure (RCAC^a^; range 1-5) and group			T0 (baseline)		T1 (directly after the intervention)										T2 (3 months later)						
			Value, mean		Value, mean (95% CI)		*P* value			Effect size (Cohen *d*)			Value, mean (95% CI)				*P* value			Effect size (Cohen *d*)	
**Total mean score**								.30			0.20							.22			0.24
	IG	3.33		3.17 (3.01-3.32)								3.07 (2.90-3.23)									
	CG	3.29		3.28 (3.13-3.43)								3.20 (3.05-3.36)									
**Fertility potential**								.19			0.25							.26			0.22
	IG	3.83		3.50 (3.21-3.78)								3.40 (3.11-3.69)									
	CG	3.95		3.76 (3.48-4.04)								3.63 (3.35-3.92)									
**Partner disclosure**								.42			−0.15							.38			−0.17
	IG	3.24		3.15 (2.87-3.44)								2.99 (2.69-3.29)									
	CG	2.95		2.99 (2.71-3.27)								2.80 (2.51-3.09)									
**Child’s health**								.11			0.30							.003			0.576
	IG	3.25		3.17 (2.86-3.47)								2.91 (2.60-3.22)									
	CG	3.49		3.52 (3.22-3.82)								3.59 (3.28-3.90)									
**Personal health**								.46			0.14							.74			0.06
	IG	3.30		3.25 (2.99-3.50)								3.22 (2.96-3.49)									
	CG	3.36		3.38 (3.13-3.64)								3.29 (3.03-3.55)									
**Acceptance**								.64			−0.09							.75			−0.06
	IG	3.12		2.98 (2.66-3.31)								2.86 (2.53-3.19)									
	CG	2.88		2.87 (2.55-3.20)								2.79 (2.45-3.12)									
**Becoming pregnant**								.46			0.14							.61			0.10
	IG	3.25		3.02 (2.80-3.25)								3.10 (2.86-3.34)									
	CG	3.10		3.14 (2.93-3.36)								3.18 (2.96-3.41)									

^a^RCAC: Reproductive Concerns After Cancer.

#### Secondary Outcomes

There was a significant difference between IG and CG on the secondary outcome cancer-related fertility knowledge, where participants in the IG had better self-rated knowledge than controls at both follow-up points (T1: mean score 2.81 vs 2.54; *P*=.05; ES=0.35 and T2: mean score 2.75 vs 2.38; *P*=.01; ES=0.42). For all other outcome measures, there were no statistically significant differences between groups at T1 or T2 (Table 3).

**Table 3:** Mean group difference on secondary outcome measures at baseline, after the intervention, and 3 months after the intervention (N=124).

Outcome subscale (range)		T0 (baseline)			T1 (12 weeks; directly after the intervention)					T2 (24 weeks; 3-month follow-up)			
		IG^a^ (n=64), mean (SD)	CG^b^ (n=60), mean (SD)	IG (n=50), mean (SD)		CG (n=58), mean (SD)	*P* value^c^	Effect size^d^	IG (n=48), mean (SD)		CG (n=53), mean (SD)	*P* value^c^	Effect size^d^
HADS^e^ anxiety (0-21)		9.84 (4.45)	8.58 (4.40)	8.67 (4.59)		8.40 (4.46)	.76	0.06	8.71 (3.77)		7.73 (4.58)	.24	0.22
HADS depression (0-21)		5.35 (3.61)	4.76 (4.01)	5.61 (3.85)		4.36 (3.62)	.09	0.33	5.24 (3.87)		3.96 (4.05)	.11	0.33
EORTC-QLQ-C30^f^ sum score (0-100)		73.34 (16.80)	76.08 (18.58)	75.06 (17.00)		77.35 (19.40)	.52	0.13	78.01 (16.28)		79.59 (18.15)	.65	0.09
Fertility self-efficacy (1-4)		3.09 (0.75)	3.30 (0.64)	3.21 (0.66)		3.20 (0.66)	.95	0.01	3.13 (0.78)		3.31 (0.67)	.24	0.26
**Fertility knowledge**													
	General (1-4)	3.58 (0.55)	3.50 (0.74)	3.51 (0.68)		3.49 (0.75)	.87	0.03	3.55 (0.57)		3.50 (0.59)	.69	0.07
	Cancer related (1-4)	2.65 (0.82)	2.60 (0.73)	2.81 (0.70)		2.54 (0.70)	.05	0.35	2.75 (0.76)		2.38 (0,7)	.01	0.48

^a^IG: intervention group.

^b^CG: control group.

^c^*t* test (2-tailed).

^d^Cohen *d* = (mean_2_ – mean_1_)/baseline SD_pooled_.

^e^HADS: Hospital Anxiety and Depression Scale.

^f^EORTC-QLQ-C30: European Organization for Research and Treatment of Cancer Quality of Life Questionnaire.

#### Self-perceived Change in Problems Regarding Fertility After Cancer

At the postintervention evaluation (T1), participants in the IG completed a single item on self-perceived change in problems regarding fertility after cancer. Most of those who completed T1 (31/50, 62%) thought their problems had been alleviated: 30% (15/50 participants) improved a little, 22% (11/50 participants) improved, and 10% (5/50 participants) improved a lot. Of the 50 participants, 18 (36%) felt that their problems had not changed and 1 (2%) experienced a worsening situation and commented that this was not because of the program.

## Discussion

### Principal Findings

This study invited young adults with cancer who had reported fertility distress in a population-based survey to test the efficacy of a psychoeducational intervention, the Fex-Can Fertility program. This study aimed to determine the capacity of this web-based, self-help program in alleviating fertility distress as measured using the RCAC scale. Assessment at the 3-month follow-up after the end of the program showed significant differences in one out of six dimensions of the RCAC scale, child’s health, where the IG had less distress than the CG. Regarding the secondary outcome of cancer-related fertility knowledge, the IG reported better knowledge than the CG at both the directly postintervention and at the 3-month follow-up. ESs were small to moderate, with a more pronounced effect at the 3-month follow-up. Subgroup analyses assessing the possible interaction effect of time and group, adherence, and baseline RCAC scores on the main outcome measure did not substantially alter the results.

### Comparison With Previous Work

The results indicating a moderate effect on distress related to genetic risks for offspring and knowledge about fertility after cancer were expected, in the sense that the program contained clear information on these topics. Previous research has found that patients value reliable information and honest communication in health care [58]. One explanation for the effect on the dimension child’s health may be that the program provided information that for most cancer diagnoses, the genetic risks involved were small and the amount of uncertainty was low. This knowledge probably calmed participants’ worries more than, for instance, learning about the impact of treatment on fertility. The fertility potential is difficult or impossible to exactly predict at the individual level [2]. Similarly, the possibilities of accessing and succeeding with assisted reproductive technologies are concerns where the program might have sustained, or even introduced, a high level of uncertainty. The information provided concerning fertility potential may also have been perceived as negative, as fertility preservation options and assisted reproductive techniques may not have been available for many participants based on female gender, age, diagnosis, or treatment regimen [59]. The same reasoning could apply to, for instance, the dimension personal health, as it contained potentially distressing information on the unpredictable risk of recurrence and potentially new information on harmful, but possibly preventable, late effects of cancer treatment; for example, cardiovascular disease. The intervention showed no effect on fertility self-efficacy, health-related quality of life, and emotional distress.

### Adherence and Activity in the Program Were Not Related to Effect

The concept of adherence to eHealth interventions is contested because of a lack of agreement on whether reported measures really refer to use leading to intended effects or simply to use of any kind [22]. In this study, a priori measures of adherence were not established but discussed by the research team at the beginning of the analysis process. To ensure validity, measures of adherence were determined based on the theoretical working mechanisms of the intervention, as suggested in the literature [22]. As in many psychosocial and eHealth interventions, the researchers could not determine an exact cutoff for *high use* a priori, despite previous feasibility testing. There is no theoretical definition of the intended *dose* to achieve a clinically meaningful effect. Whether participants with high use levels also benefited more from the program is, therefore, not completely clear. Qualitative interviews with a subsample of participants in the IG suggested that some individuals who had been relatively inactive found the program, or parts of it, helpful [60]. This may explain why there were no clear results for the models investigating dose or adherence. It could also be that some high-level users became more anxious from the program as they became more aware of treatment-related fertility risks or their own health or because they were in vain searching for comforting information. Some studies suggest that for certain individuals, turning to counseling in health care or looking for support on social media may coincide with an aggravation of distress [61,62]. Considering that the overall use of the program was limited, it can also be questioned whether what was defined a posteriori as *high use* (at least 20 minutes spent on the website, opening half of the modules, and one measure of interactivity in a period of 12 weeks) corresponded to a level of use or intensity that would produce an independent effect.

This study had very small formal dropout rates in the postintervention follow-up, that is, most participants returned questionnaires at both follow-up points. In an intention-to-treat manner, surveys were sent via mail to all patients who were randomized, regardless of their activity level. This means that participants who had not been very active in the program and some who had not even logged on to the website responded to postintervention surveys and were counted as completers alongside their more dedicated counterparts, possibly *washing out* some potential effects of the intervention. However, subgroup analyses based on activity did not show that participants with higher activity benefited more from the program.

### Methodological Considerations

Strengths of this study included having a thoroughly prepared, theory-based intervention designed with a participatory approach [31], reaching the whole intended population for eligibility assessment with a validated instrument [38,39], and retaining high response rates throughout the study. However, we wanted to focus on some limitations contributing to why the results must be interpreted with caution.

RCTs are usually considered the gold standard for scientific evidence. However, in social and psychological interventions, especially eHealth interventions, conditions are not fully controlled, as double-blinding is not possible. The researchers cannot influence what type of accessory support either the IG or the CG has access to, and substantial self-help information is readily available on websites via social or traditional media. This may lead to an inconclusive assessment of intervention effects. Furthermore, there are various sources of bias introduced by design choices, such as not having a set standard for adherence; for example, homework or a minimum assignment for participants. Although evidence for efficacious web-based psychoeducational interventions remains weak [19], reviews on internet-based cognitive behavioral therapy (ICBT) show that therapist-led interventions have larger effects than self-guided programs [63] and are potentially as effective as face-to-face therapy [63,64]. Generally, effective ICBT programs are characterized by a relatively firm structure and limited uptake [65]. The present intervention format was more flexible, and adherence according to the chosen definition did not seem to be associated with an improved effect on the main outcome measure, suggesting that the mechanisms of impact require further investigation and may not be the same as those for ICBT.

To the best of our knowledge, dimensions of the RCAC scale have been used as intervention outcome measures in only one previously published study. A study by Su et al [14] assessed 2 dimensions of the RCAC scale—fertility potential and becoming pregnant—as part of a comprehensive survivorship care plan for women with breast cancer, in which the proportion of participants having improved (moving from >3 to ≤3 on the subscale mean) was statistically significantly larger in the IG than in the CG. In the present study, all dimensions of RCAC were used. It remains unclear whether the instrument is sensitive enough to detect meaningful changes and what the appropriate clinical cutoff level would be. Indeed, when asked in the postintervention survey, 62% (31/50) of the participants in the IG stated that they had improved during the intervention period, but this was not reflected in ratings on the main outcome measure. Active participation in the program was generally low, which may partially explain the lack of group differences. Finally, a contributing factor to not finding more pronounced effects is that despite designing contents of the program to encompass all known aspects of fertility-related distress, the chosen outcome measures may not have adequately captured the change induced by the intervention. Part of the theoretical framework for the intervention relied on efforts to enhance participants’ self-efficacy and satisfaction with the basic needs for competence, relatedness, and autonomy. Drawing on the study by Pingree et al [29], we expected that before affecting distal or long-term outcomes, such as quality of life, an intervention may influence intermediate outcome measures, such as basic need satisfaction. The analyses failed to detect statistically significant differences in most outcome measures, including fertility-related self-efficacy. As no measure of basic need satisfaction or other types of motivational measure had been included in the evaluation of the intervention, we were unable to draw conclusions on intermediate outcomes. However, participating in the program did not seem to have produced any adverse outcomes, and most participants stated in both the postintervention survey and qualitative interviews that their distress had been reduced [60], suggesting there was at least some perceived benefit from the intervention.

### Conclusions

This web-based psychoeducational intervention for young adults diagnosed with cancer had little overall effect on fertility-related distress. Small to moderate effects could be seen on cancer-related fertility knowledge and the level of concern for future children’s health. Further research on the mechanisms of impact is required to determine for whom the Fex-Can program or similar interventions may constitute an appropriate individualized support.

### Clinical Implications

The Fex-Can Fertility program could be useful for improving knowledge about fertility and reducing concerns about genetic risks following cancer. The automated, flexible, and partially tailored design of the intervention makes it a convenient tool in clinical care. It appears safe to use because no adverse effects were reported and most participants reported subjective improvement in their concerns.

## Appendix

Multimedia Appendix 1 Study-specific instruments.

Multimedia Appendix 2 Significant differences in subgroup analyses based on activity level.

Multimedia Appendix 3 Subgroup analyses based on baseline levels of fertility distress.

Multimedia Appendix 4 Subgroup analyses based on activity level.

Multimedia Appendix 5 Reproductive Concerns After Cancer dimension 5 (acceptance). Subgroup analyses according to baseline levels.

Multimedia Appendix 6 CONSORT eHEALTH Checklist (V 1.6.1).

## Abbreviations

CG: control group
CONSORT: Consolidated Standards of Reporting Trials
ES: effect size
Fex-Can: Fertility and Sexuality following Cancer
ICBT: internet-based cognitive behavioral therapy
IG: intervention group
RCAC: Reproductive Concerns After Cancer
RCT: randomized controlled trial
SDT: self-determination theory

## Data Availability

The software application code and the original data set are available upon reasonable request.
