# Supplementary material for: Efficacy of a Web-Based Psychoeducational Intervention for Young Adults With Fertility-Related Distress Following Cancer (Fex-Can): Randomized Controlled Trial
Source: JMIR Cancer. 2022 Mar 29;8(1):e33239. doi: 10.2196/33239 (PMC9006131; doi:10.2196/33239)
Supplement: Multimedia Appendix 1 [file cancer_v8i1e33239_app1.docx]

Study-specific instruments, translated from original Swedish

| **SELF-EFFICACY FERTILITY** |
| --- |
| Response alternatives: 1=strongly disagree to 4=strongly agree, and one additional alternative ”Not relevant” |
| **I feel confident that I can..** |
| cope with negative thoughts and feelings in relation to my reproductive ability |
| cope with meeting friends or relatives who are pregnant |
| cope with feelings of anger or hostility towards others who have formed a family |
| tell a (future) partner that it is (may be) difficult for me to have children |
| tell other people I’m concerned about my reproductive ability |
| *have a positive attitude towards starting a family** |
|  |
| **FERTILITY-RELATED KNOWLEDGE** |
| Response alternatives: 1= disagree completely to 4=agree completely |
| **"I have good knowledge regarding..”** |
| General |
| the menstrual cycle and when a pregnancy can occur |
| the importance of age for the ability to have children |
| the importance of lifestyle for the ability to have children |
| the chance of becoming pregnant at one attempt |
| Cancer related |
| how the cancer treatment affects my body |
| the effect of cancer and cancer treatments on reproductive ability |
| if cancer or cancer treatments can affect a pregnancy or childbirth |
| if cancer or cancer treatments can affect the health of one’s future children |
| different types of assisted reproduction (e.g. insemination and IVF) |
| the possibility of adoption following cancer |

* Item excluded from mean score for Fertility self-efficacy due to low correlation with remaining items
