# Supplementary figures and images for: Efficacy of a Web-Based Psychoeducational Intervention for Young Adults With Fertility-Related Distress Following Cancer (Fex-Can): Randomized Controlled Trial
Source: JMIR Cancer. 2022 Mar 29;8(1):e33239. doi: 10.2196/33239 (PMC9006131; doi:10.2196/33239)

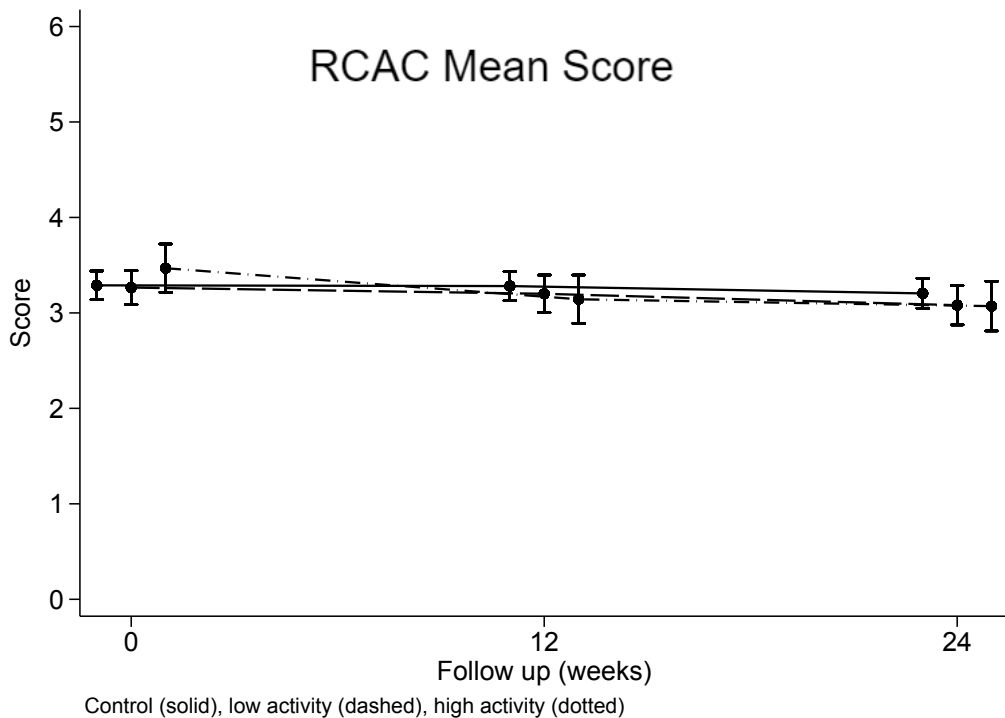

## Dimension 1: Fertility potential

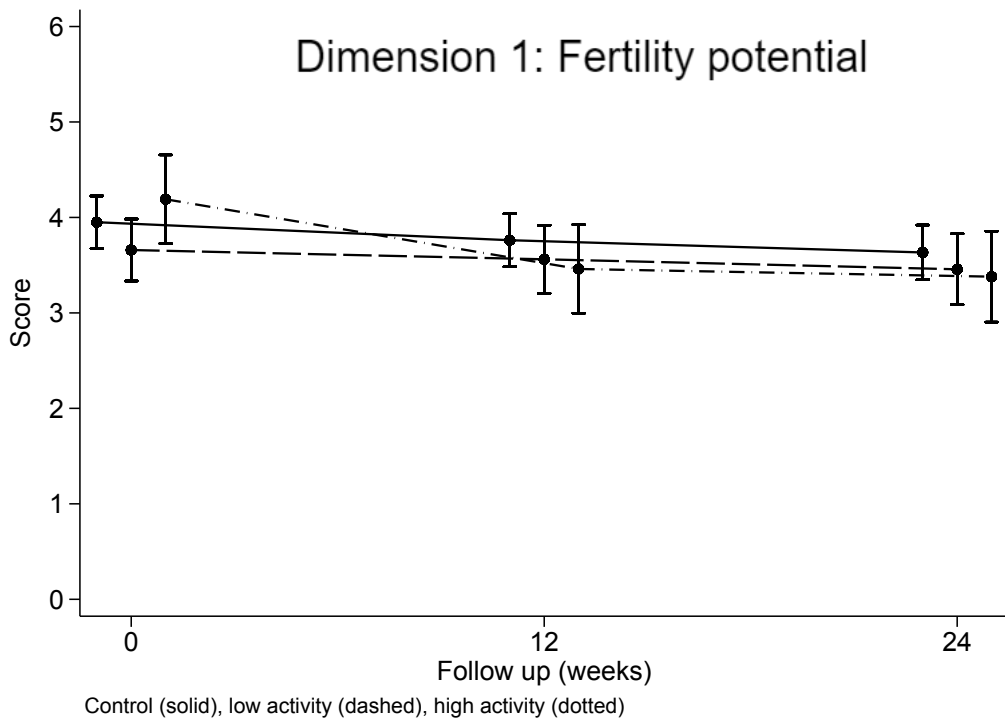

### Dimension 3: Child' health

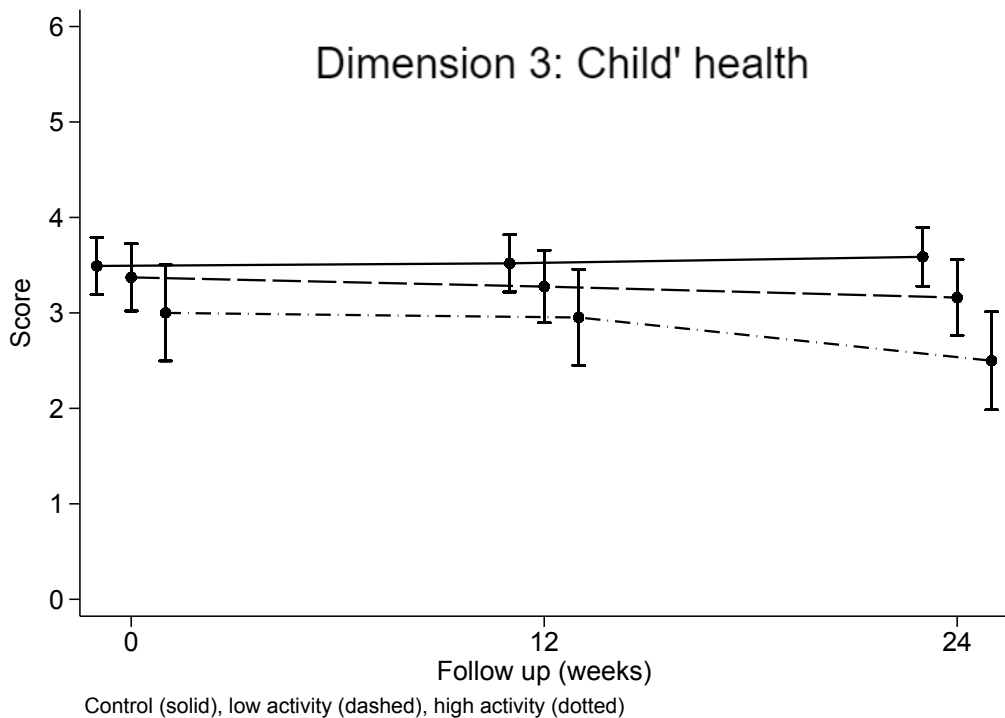

## Dimension 5: Acceptance

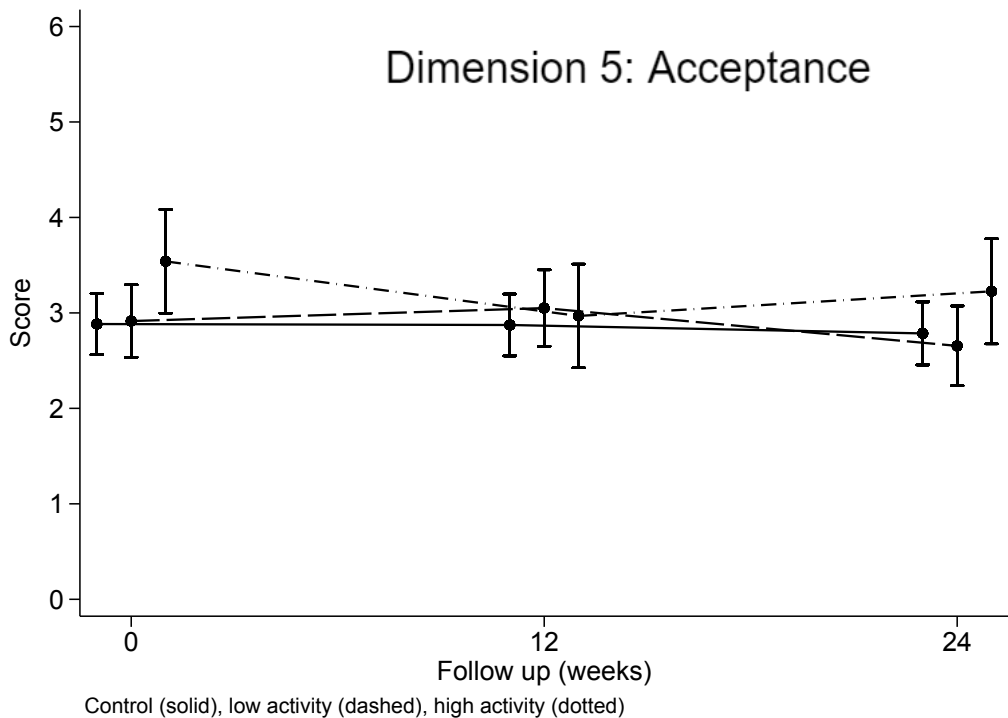

Supplement: Multimedia Appendix 2 [file cancer_v8i1e33239_app2.pdf]

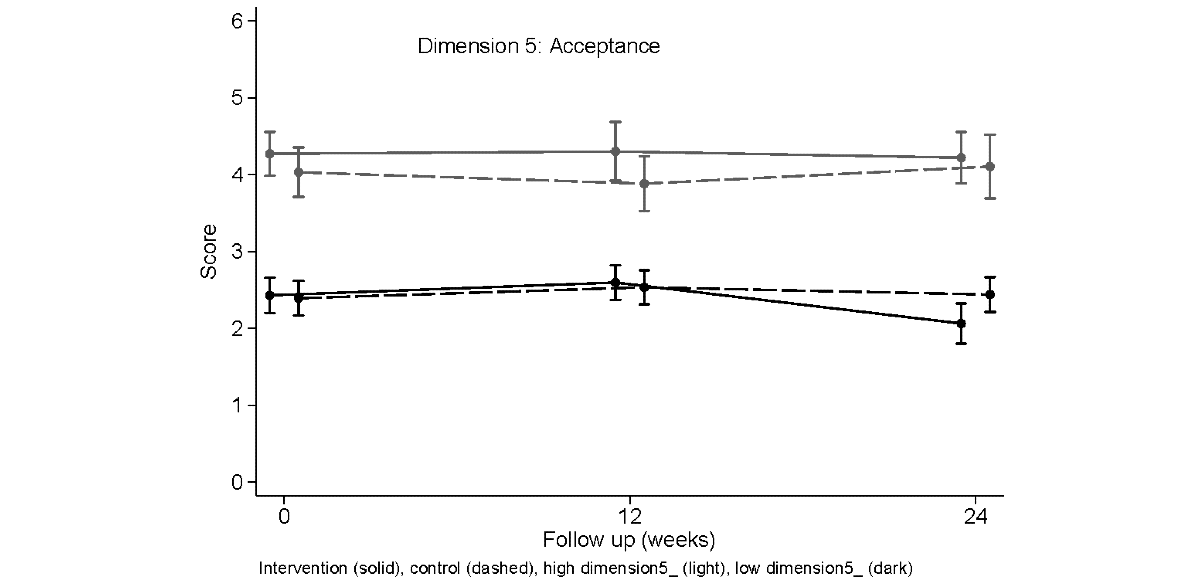

Supplement: Multimedia Appendix 5 [file cancer_v8i1e33239_app5.png]
