# Supplementary material for: Efficacy of a Web-Based Psychoeducational Intervention for Young Adults With Fertility-Related Distress Following Cancer (Fex-Can): Randomized Controlled Trial
Source: JMIR Cancer. 2022 Mar 29;8(1):e33239. doi: 10.2196/33239 (PMC9006131; doi:10.2196/33239)
Supplement: Multimedia Appendix 3 [file cancer_v8i1e33239_app3.docx]

Table 3b. Difference in mean values between groups over time (Linear mixed models with random intercept; group and time interaction). Subgroup analyses based on baseline levels of fertility distress.

|  | **Subgroup analyses according to baseline levels of fertility distress: IG Low RCAC vs CG Low RCAC** | | | | | | | | | | |
| --- | --- | --- | --- | --- | --- | --- | --- | --- | --- | --- | --- |
| Outcome (range 1-5) |  |  | **T0** | **T1** | | | | **T2** | | | |
| **RCAC** | **Group** | **N^[[1]](#footnote-1)^** | **Mean** | **Mean** | **95% CI** | **p^[[2]](#footnote-2)^** | **ES^[[3]](#footnote-3)^** | **Mean** | **95% CI** | **P^1^** | **ES^2^** |
| **Mean Score** | IG | 141 | 3.21 | 3.11 | 2.99-3.24 | 0.62 | 0.10 | 2.95 | 2.81-3.09 | 0.11 | 0.34 |
|  | CG | 141 | 3.17 | 3.16 | 3.03-3.29 |  |  | 3.10 | 2.97-3.24 |  |  |
| **Fertility Potential** | IG | 83 | 2.84 | 2.76 | 2.54-2.99 | 0.21 | 0.34 | 2.61 | 2.39-2.84 | 0.08 | 0.49 |
|  | CG | 76 | 2.97 | 2.97 | 2.74-3.20 |  |  | 2.91 | 2.67-3.14 |  |  |
| **Partner Disclosure** | IG | 115 | 2.67 | 2.60 | 2.38-2.83 | 0.91 | 0.02 | 2.53 | 2.30-2.76 | 0.67 | -0.10 |
|  | CG | 138 | 2.59 | 2.62 | 2.42-2.82 |  |  | 2.46 | 2.25-2.67 |  |  |
| **Child’s health** | IG | 109 | 2.50 | 2.54 | 2.31-2.77 | 0.30 | 0.27 | 2.42 | 2.19-2.65 | 0.30 | 0.27 |
|  | CG | 83 | 2.75 | 2.72 | 2.47-2.98 |  |  | 2.70 | 2.43-2.98 |  |  |
| **Personal health** | IG | 123 | 3.01 | 2.91 | 2.71-3.11 | 0.29 | 0.23 | 2.78 | 2.57-2.99 | 0.72 | 0.08 |
|  | CG | 114 | 2.81 | 3.06 | 2.86-3.25 |  |  | 2.83 | 2.62-3.05 |  |  |
| **Acceptance** | IG | 109 | 2.43 | 2.60 | 2.37-2.82 | 0.70 | -0.09 | 2.06 | 1.80-2.32 | 0.03 | 0.53 |
|  | CG | 125 | 2.39 | 2.53 | 2.31-2.76 |  |  | 2.44 | 2.21-2.67 |  |  |
| **Becoming pregnant** | IG | 128 | 2.80 | 2.85 | 2.68-3.01 | 0.93 | -0.02 | 2.79 | 2.61-2.96 | 0.15 | 0.33 |
|  | CG | 130 | 2.69 | 2.84 | 2.67-3.00 |  |  | 2.97 | 2.79-3.14 |  |  |

Table 3b. (cont.) Difference in mean values between groups over time (Linear mixed models with random intercept; group and time interaction). Subgroup analyses based on baseline levels of fertility distress.

|  | **Subgroup analyses according to baseline levels of fertility distress: IG High RCAC vs CG High RCAC** | | | | | | | | | | |
| --- | --- | --- | --- | --- | --- | --- | --- | --- | --- | --- | --- |
| Outcome (range 1-5) |  |  | **T0** | **T1** | | | | **T2** | | | |
| **RCAC** | **Group** | **N^[[4]](#footnote-4)^** | **Mean** | **Mean** | **95% CI** | **P^1^** | **ES^2^** | **Mean** | **95% CI** | **P^1^** | **ES^2^** |
| **Mean Score** | IG | 18 | 4.01 | 3.95 | 3.51-4.40 | 0.32 | 0.47 | 4.01 | 3.67-4.35 | 0.86 | 0.09 |
|  | CG | 26 | 3.96 | 3.87 | 3.62-4.13 |  |  | 3.93 | 3.63-4.23 |  |  |
| **Fertility Potential** | IG | 79 | 4.56 | 4.30 | 4.06-4.54 | 0.46 | 0.20 | 4.48 | 4.20-4.75 | 0.86 | -0.05 |
|  | CG | 93 | 4.56 | 4.42 | 4.21-4.64 |  |  | 4.44 | 4.21-4.68 |  |  |
| **Partner Disclosure** | IG | 48 | 4.59 | 4.44 | 4.11-4.76 | 0.87 | 0.06 | 4.57 | 4.19-4.95 | 0.55 | -0.25 |
|  | CG | 31 | 4.73 | 3.37 | 3.03-4.38 |  |  | 4.39 | 3.95-4.83 |  |  |
| **Child’s health** | IG | 54 | 4.34 | 4.20 | 3.89-4.52 | 0.78 | 0.08 | 4.30 | 3.89-4.71 | 0.72 | 0.13 |
|  | CG | 86 | 4.34 | 4.26 | 4.01-4.51 |  |  | 4.38 | 4.13-4.64 |  |  |
| **Personal health** | IG | 40 | 4.44 | 4.19 | 3.88-4.49 | 0.85 | 0.06 | 4.26 | 3.95-4.58 | 0.76 | -0.10 |
|  | CG | 55 | 4.37 | 4.23 | 3.93-4.52 |  |  | 4.20 | 3.92-4.47 |  |  |
| **Acceptance** | IG | 52 | 4.27 | 4.30 | 3.92-4.69 | 0.12 | -0.59 | 4.22 | 3.89-4.55 | 0.67 | -0.16 |
|  | CG | 42 | 4.03 | 3.88 | 3.53-4.24 |  |  | 4.10 | 3.69-4.52 |  |  |
| **Becoming pregnant** | IG | 34 | 4.33 | 4.02 | 3.60-4.43 | 0.66 | 0.21 | 4.39 | 4.00-4.77 | 0.37 | -0.43 |
|  | CG | 39 | 4.34 | 4.13 | 3.84-4.42 |  |  | 4.15 | 3.80-4.49 |  |  |

1. Total number of observations [↑](#footnote-ref-1)
2. Of the difference between group means at the point estimate. [↑](#footnote-ref-2)
3. Calculated by dividing the difference of the means by the variance of the residual [↑](#footnote-ref-3)
4. Total number of observations [↑](#footnote-ref-4)
