# Supplementary material for: Efficacy of a Web-Based Psychoeducational Intervention for Young Adults With Fertility-Related Distress Following Cancer (Fex-Can): Randomized Controlled Trial
Source: JMIR Cancer. 2022 Mar 29;8(1):e33239. doi: 10.2196/33239 (PMC9006131; doi:10.2196/33239)
Supplement: Multimedia Appendix 4 [file cancer_v8i1e33239_app4.docx]

Table 3c. Difference in mean values between groups over time (Linear mixed models with random intercept; group and time interaction). Subgroup analyses based on dose/adherence (activity).

| Outcome (range 1-5) |  | **T0** | **T1** | | | **T2** | | |
| --- | --- | --- | --- | --- | --- | --- | --- | --- |
| **RCAC** | **Group^[[1]](#footnote-1)^** | **Mean** | **Mean** | **CI 95%** | **p^[[2]](#footnote-2)^** | **Mean** | **CI 95%** | **P^2^** |
| **Mean Score** | High | 3.47 | 3.14 | 2.89-3.40 | .02 | 3.07 | 2.81-3.33 | <.001 |
|  | Low | 3.27 | 3.20 | 3.00-3.40 |  | 3.08 | 2.87-3.29 |  |
|  | Control | 3.29 | 3.28 | 3.13-3.43 |  | 3.20 | 3.05-3.36 |  |
| **Fertility Potential** | High | 4.19 | 3.46 | 3.00-3.92 | .009 | 3.38 | 2.90-3.86 | <.001 |
|  | Low | 3.66 | 3.56 | 3.20-3.92 |  | 3.46 | 3.09-3.83 |  |
|  | Control | 3.95 | 3.76 | 3.48-4.04 |  | 3.63 | 3.35-3.92 |  |
| **Partner Disclosure** | High | 3.52 | 3.27 | 2.80-3.74 | .71 | 3.19 | 2.71-3.67 | .20 |
|  | Low | 3.10 | 3.10 | 2.74-3.47 |  | 2.88 | 2.50-3.26 |  |
|  | Control | 2.95 | 2.99 | 2.71-3.27 |  | 2.80 | 2.51-3.09 |  |
| **Child’s health** | High | 3 | 2.95 | 2.45-3.46 | .92 | 2.50 | 1.98-3.01 | .04 |
|  | Low | 3.37 | 3.28 | 2.90-3.66 |  | 3.16 | 2.76-3.56 |  |
|  | Control | 3.49 | 3.52 | 3.22-3.82 |  | 3.59 | 3.28-3.90 |  |
| **Personal health** | High | 3.03 | 3.13 | 2.70-3.55 | .71 | 2.91 | 2.48-3.34 | .82 |
|  | Low | 3.43 | 3.30 | 2.98-3.62 |  | 3.40 | 3.07-3.74 |  |
|  | Control | 3.36 | 3.38 | 3.13-3.64 |  | 3.29 | 3.03-3.55 |  |
| **Acceptance** | High | 3.54 | 2.76 | 2.09-3.43 | .006 | 3.23 | 2.68-3.78 | .07 |
|  | Low | 2.91 | 3.05 | 2.65-3.45 |  | 2.65 | 2.24-3.07 |  |
|  | Control | 2.88 | 2.87 | 2.55-3.20 |  | 2.79 | 2.45-3.12 |  |
| **Becoming pregnant** | High | 3.52 | 3.08 | 2.72-3.44 | .13 | 3.22 | 2.85-3.60 | .42 |
|  | Low | 3.12 | 3.01 | 2.72-3.30 |  | 3.04 | 2.73-3.34 |  |
|  | Control | 3.10 | 3.14 | 2.93-3.36 |  | 3.18 | 2.96-3.41 |  |

1. Total number of observations: High N=4; Low N=150; Control N=180 [↑](#footnote-ref-1)
2. P-value of the difference. Effect sizes not calculated due to more than two groups being compared. [↑](#footnote-ref-2)
