# Supplementary material for: Efficacy of a Web-Based Psychoeducational Intervention for Young Adults With Fertility-Related Distress Following Cancer (Fex-Can): Randomized Controlled Trial
Source: JMIR Cancer. 2022 Mar 29;8(1):e33239. doi: 10.2196/33239 (PMC9006131; doi:10.2196/33239)
Supplement: Multimedia Appendix 6 [file cancer_v8i1e33239_app6.pdf]

CONSORT-EHEALTH Checklist V1.6.2 Report	Manuscript Number	33239
**(based on CONSORT-EHEALTH V1.6), available at [http://tinyurl.com/consort-ehealth-v1-6].**		
**Date completed**		
12/17/2021 17:18:18		
**by**		
Claire Micaux		
Efficacy of Fex-Can Fertility, a web-based psychoeducational intervention for young adults with fertility-related distress following cancer – a randomized controlled trial.		
TITLE		
**1a-i) Identify the mode of delivery in the title**		
"a web-based psychoeducational intervention"		
**1a-ii) Non-web-based components or important co-interventions in title**		
Not applicable since there were no such components		
**1a-iii) Primary condition or target group in the title**		
"for young adults with fertility-related distress following cancer"		
ABSTRACT		
**1b-i) Key features/functionalities/components of the intervention and comparator in the METHODS section of the ABSTRACT**		
"This Randomized Controlled Trial (RCT) evaluated a 12-week web-based, automated self-help intervention for fertility-related distress following cancer, The Fex-Can Fertility. Individuals were identified via Swedish national quality registries and those reporting fertility-related distress 1.5 years post-diagnosis were invited. A total of 100 women and 24 men (age 19-40) answered self-administered surveys at baseline (T0), directly post-intervention (T1) and three months later (T2). Main outcome was fertility-related distress measured with the six-dimension Reproductive Concerns After Cancer (RCAC) scale. Secondary outcomes were health-related quality of life (EORTC-QLQ-C30), emotional distress (HADS), fertility-related knowledge and fertility self-efficacy. In addition, the intervention group (IG) reported their self-perceived change in problems related to fertility after cancer (T1). T-tests and linear mixed models including intention-to-treat and subgroup analyses were performed to compare the effects of the intervention with standard care".		
**1b-ii) Level of human involvement in the METHODS section of the ABSTRACT**		
"web-based, automated self-help intervention"		
**1b-iii) Open vs. closed, web-based (self-assessment) vs. face-to-face assessments in the METHODS section of the ABSTRACT**		
"Individuals were identified via Swedish national quality registries and those reporting fertility-related distress 1.5 years post-diagnosis were invited. A total of 100 women and 24 men (age 19-40) answered self-administered surveys at baseline (T0), directly post-intervention (T1) and three months later (T2)."		
**1b-iv) RESULTS section in abstract must contain use data**		
"Although 60% in the IG stated their concerns about fertility were fewer post-intervention, there were few statistically significant group differences in the main outcome (RCAC) at T1 and T2. Compared to controls, the IG rated lower distress concerning the dimension Child's health at T2 (p=0.003, ES=0.64). This difference was maintained when adding group x time interaction (intention-to-treat; p=0.003, ES=0.58). The IG also perceived better cancer-related fertility knowledge at T1/T2 (p=0.050, ES=0.35/p=0.013, ES=0.42) than did the control group. Subgroup analyses based on dose/adherence and baseline RCAC scores did not substantially alter the results. Overall utilization of the web-based program was low."		
**1b-v) CONCLUSIONS/DISCUSSION in abstract for negative trials**		
"The lack of group differences in other dimensions of fertility distress and related secondary outcomes contrasted with reports on self-perceived improvement post-intervention. "		
INTRODUCTION		
**2a-i) Problem and the type of system/solution**		

"Psychosocial interventions for cancer survivors, which may or may not include web-based components, often have a broad scope [13], and are referred to as ‘survivorship care plans’ [14], self-management interventions [15] or multidimensional programs [16, 17]. There is a shortage of interventions targeting both medical and psychosocial concerns about fertility and parenthood following cancer."

2a-ii) Scientific background, rationale: What is known about the (type of) system

"In the past decade, eHealth has exploded as a research and clinical discipline, and the number of psychosocial and psychological interventions has risen. A number of reviews have pointed out the complex nature of eHealth interventions as well as challenges involved in their testing and implementation [19-21]. For example, there is limited evidence concerning dose and adherence measures [22]"

Does your paper address CONSORT subitem 2b?

"The aim of the present study was to test the efficacy of the Fex-Can intervention in reducing fertility-related distress and related psychosocial outcomes in young adults with cancer.
Specific research questions:
1)Is the Fex-Can Fertility program superior to standard care in reducing fertility distress directly after the end of the program, and 3 months later, respectively?
2)Does the Fex-Can Fertility program increase fertility self-efficacy and fertility-related knowledge, reduce emotional distress or improve health-related quality of life, compared to standard care?
3)Do baseline levels of fertility distress predict the effect of the program over time?
4)Does dose, i.e., the uptake and adherence to the program, influence the change over time in fertility distress ratings?"

METHODS

3a) CONSORT: Description of trial design (such as parallel, factorial) including allocation ratio

The Fex-Can project encompasses a national cohort study [38] with an embedded randomized controlled trial (RCT) including participants with self-reported distress/dysfunction at baseline [39]. The Fex-Can web-based psychoeducational program was offered in two versions; Fex-Can Sex and Fex-Can Fertility, the latter being evaluated in the present study. Detailed description of the study design is available in two published study protocols [38, 39] and is briefly described below.
Allocation (1-1 ratio) to either intervention (IG) or control group (CG) was performed by an external statistician uninvolved in the data collection process, by stratified block randomization taking account of sex and diagnosis. Due to the design of the intervention a placebo condition was not possible and neither participants nor researchers could be blinded to group allocation. Participants were considered lost to follow-up only if they for any reason did not return post-intervention questionnaires; hence no pattern of attrition was determined post-randomization"

3b) CONSORT: Important changes to methods after trial commencement (such as eligibility criteria), with reasons

There was a change in the number of primary outcome measures due to recent publications validating the use of the RCAC scale as six separate subscales rather than one mean score.

3b-i) Bug fixes, Downtimes, Content Changes

Not relevant since it did not happen

4a) CONSORT: Eligibility criteria for participants

"The sample was drawn from a cohort of 1499 individuals diagnosed with breast, cervical, ovarian or testicular cancer, lymphoma, or CNS tumor between 2016-2017, approximately 1.5 years prior to the start of the study. The time frame was chosen to approach people who were likely to have finished primary treatment but still close enough to diagnosis to be in need of psychosocial support. Eligible participants were identified using Swedish national quality registries and all people in the intended age bracket (18-39 at diagnosis) were approached for a longitudinal cohort study. Individuals reporting fertility distress at the baseline assessment were invited to the Fex-Can Fertility trial.

Eligibility
Respondents scoring ≥4 on at least one subscale of the Reproductive Concerns After Cancer (RCAC) scale [4] were eligible for the RCT."

4a-i) Computer / Internet literacy

Not relevant - we assumed only people with a certain level of cimputer literacy would consent to participate in a fully web-based intervention study. In addition, the target group were young adults in one of the most computerized countries in the world.

**4a-ii) Open vs. closed, web-based vs. face-to-face assessments:**		
"Eligible participants were identified using Swedish national quality registries and all people in the intended age bracket (18-39 at diagnosis) were approached for a longitudinal cohort study with a letter containing a survey sent to their population registration address. The survey could be completed on paper or online."		
**4a-iii) Information giving during recruitment**		
"Individuals reporting fertility distress at the baseline assessment were invited to the Fex-Can Fertility trial and had to send a signed form back granting their consent to participate in the RCT."		
**4b) CONSORT: Settings and locations where the data were collected**		
Not applicable, data were collected all over Sweden.		
**4b-i) Report if outcomes were (self-)assessed through online questionnaires**		
"The survey could be completed on paper or online"		
**4b-ii) Report how institutional affiliations are displayed**		
**5) CONSORT: Describe the interventions for each group with sufficient details to allow replication, including how and when they were actually administered**		
**5-i) Mention names, credential, affiliations of the developers, sponsors, and owners**		
**5-ii) Describe the history/development process**		
"The development, design, contents and structure of the intervention have been described in detail in previous publications [31, 37, 44]"		
**5-iii) Revisions and updating**		
Not applicable since no revisions were made during the trial		
**5-iv) Quality assurance methods**		
The contents of the intervention went through expert review, which is described in previous publications		
**5-v) Ensure replicability by publishing the source code, and/or providing screenshots/screen-capture video, and/or providing flowcharts of the algorithms used**		
The source code will be provided upon reasonable request.		
**5-vi) Digital preservation**		
**5-vii) Access**		
"The development, design, contents and structure of the intervention have been described in detail in previous publications [31, 37, 44]. "		
**5-viii) Mode of delivery, features/functionalities/components of the intervention and comparator, and the theoretical framework**		

Intervention<br>"The intervention was a 12-week, web-based psycho-educational program. The Fex-Can Fertility program was organized in six successive modules with informational material, texts and exercises aiming at developing competence and facilitating behavior change through a sound balance between change and acceptance strategies. The modules covered known aspects of fertility distress [4] and were entitled: Fertility after cancer, Handling anxiety, Trying to have children after cancer, My own health and my child's health, Not being able to have biological children, and Relationships. Contents are described in detail in a doctoral thesis aiming for a process and outcome evaluation of the Fex-Can Fertility intervention [44]. The mode of delivery was conceived to facilitate satisfaction of participants' basic needs according to self-determination theory [33]. It was assumed such theoretical orientation would enhance positive health outcomes such as self-efficacy and health-related quality of life [29]. Nuanced information and reliable facts were intended to leverage participants' competence. Written and filmed survivor stories as well as interactive quizzes and a discussion forum were included with the goal of helping participants find strategies to handle their concerns surrounding fertility and family-building after cancer by strengthening autonomy and relatedness. The development, design, contents and structure of the intervention have been described in detail in previous publications [31, 37, 44]. The discussion forum was moderated by one of the research partners [31] and by a member of the research team with clinical expertise in psychology or nursing. Adherence was defined using quantitative activity parameters retrieved from the website system data."		
**5-ix) Describe use parameters**		
"High activity was defined as: having opened at least half of the modules and spent a total of at least 20 minutes on the website (general activity), plus one of the following: having spent three minutes or more in the discussion forum, written a post in the forum, or answering 50% or more of the quizzes (interactivity). All participants who did not reach these criteria were categorized as "low activity", which could also include not having logged on to the program at all."		
**5-x) Clarify the level of human involvement**		
"The discussion forum was moderated by one of the research partners [31] and by a member of the research team with clinical expertise in psychology or nursing. "		
**5-xi) Report any prompts/reminders used**		
This is described in more detail in previous publications		
**5-xii) Describe any co-interventions (incl. training/support)**		
Not applicable since there were no co-interventions		
**6a) CONSORT: Completely defined pre-specified primary and secondary outcome measures, including how and when they were assessed**		

"Main outcome measure - Fertility distress<br>The Reproductive Concerns After Cancer Scale (RCAC) was developed for women in the US with various cancer diagnoses [4] and has been validated for women in China [47] and Sweden [48], and for men in the US [12]. The scale consists of a total score and six three-item dimensions related to fertility, pregnancy and parenthood after cancer: Fertility potential (concerns about one's ability to become a biological parent), Partner disclosure (concerns related to telling a partner about possibly impaired fertility), Child's health (concerns for a biological child's health in relation to the parent's previous cancer diagnosis and treatment, specifically genetic risks), Personal health (concerns related to fear of not being able to or living long enough to raise a child), Acceptance (the extent of reconciliation with not being fertile or not having biological children, and Becoming pregnant (concerns related to efforts involved in achieving a pregnancy). Answers are given on a five-point scale ranging from Strongly disagree (1) to Strongly agree (5), where higher scores indicate higher level of concerns. The mean of the total score as well as the mean scores for each of the six dimensions, as recommended in a validation study of the RCAC, [49] were used as primary outcomes for the Fex-Can Fertility trial.<br><br>Secondary outcome measures<br>Health-related quality of life<br>Health-related quality of life was measured with the validated [50] summary score (range 0-100) of the EORTC-QLQ-C30 version 3.0 questionnaire which is a generic instrument for all cancer diagnoses [51].<br><br>Emotional distress<br>The Hospital Anxiety and Depression Scale (HADS) is a widely used scale measuring anxiety (7 items) and depression (7 items), validated for use in cancer patients [52].<br>Scores are given on a numbered Likert scale ranging from 0 through 3 and for each subscale a total score 0-21 (with higher values indicating more anxiety/depressive symptoms) is calculated.<br><br>Fertility self-efficacy<br>Perceived confidence in one's ability to manage situations and emotions related to the threat of infertility was measured with a study-specific questionnaire based on previous research [53, 54], including six items with statements such as: "I feel confident that I can tell other people I'm concerned about my reproductive ability". All the items are available in Supplementary file 1. Answers were given on a 4-point Likert scale with alternatives ranging from completely disagree (1) to agree completely (4). An exploratory factor analysis (data not shown) indicated that one of the items was poorly correlated with the others. A mean score was calculated from the five remaining items, with higher values indicating higher levels fertility-related self-efficacy.<br><br>Fertility-related knowledge<br>The perceived level of knowledge concerning fertility issues was measured using a study-specific questionnaire developed from previous research [18] and consisting of 10 items. Answers were given on a 4-point Likert scale with alternatives ranging from completely disagree (1) to agree completely (4). Exploratory factor analysis (data not shown) on the total cohort of eligible participants indicated it was suitable to divide the scale into two domains; one for general fertility-related knowledge (4 items) and one for cancer-related fertility knowledge (6 items). Items included statements such as: "I have good knowledge regarding the menstrual cycle and when a pregnancy can occur" (general fertility knowledge) and "I have good knowledge regarding the effect of cancer and cancer treatments on reproductive ability" (cancer-related fertility knowledge). All the items are available in Supplementary file 1. Means were calculated for each subscale, with higher mean scores indicating better perceived knowledge"		
**6a-i) Online questionnaires: describe if they were validated for online use and apply CHERRIES items to describe how the questionnaires were designed/deployed**		
**6a-ii) Describe whether and how "use" (including intensity of use/dosage) was defined/measured/monitored**		
"High activity was defined as: having opened at least half of the modules and spent a total of at least 20 minutes on the website (general activity), plus one of the following: having spent three minutes or more in the discussion forum, written a post in the forum, or answering 50% or more of the quizzes (interactivity). All participants who did not reach these criteria were categorized as "low activity", which could also include not having logged on to the program at all."		
**6a-iii) Describe whether, how, and when qualitative feedback from participants was obtained**		

This is described in detail in previous publications		
**6b) CONSORT: Any changes to trial outcomes after the trial commenced, with reasons**		
Not applicable, data were collected all over Sweden.		
**7a) CONSORT: How sample size was determined**		
**7a-i) Describe whether and how expected attrition was taken into account when calculating the sample size**		
"Sample size was estimated to 128 individuals needed at follow-up, to obtain statistically significant results, assuming 80% power, medium effect size (0.5) and a significance level set at 0.05. Since the attrition between baseline and first follow-up was expected to be around 15%, we aimed to include 210 participants at baseline."		
**7b) CONSORT: When applicable, explanation of any interim analyses and stopping guidelines**		
"Main outcome measure - Fertility distress<br>The Reproductive Concerns After Cancer Scale (RCAC) was developed for women in the US with various cancer diagnoses [4] and has been validated for women in China [47] and Sweden [48], and for men in the US [12]. The scale consists of a total score and six three-item dimensions related to fertility, pregnancy and parenthood after cancer: Fertility potential (concerns about one's ability to become a biological parent), Partner disclosure (concerns related to telling a partner about possibly impaired fertility), Child's health (concerns for a biological child's health in relation to the parent's previous cancer diagnosis and treatment, specifically genetic risks), Personal health (concerns related to fear of not being able to or living long enough to raise a child), Acceptance (the extent of reconciliation with not being fertile or not having biological children, and Becoming pregnant (concerns related to efforts involved in achieving a pregnancy). Answers are given on a five-point scale ranging from Strongly disagree (1) to Strongly agree (5), where higher scores indicate higher level of concerns. The mean of the total score as well as the mean scores for each of the six dimensions, as recommended in a validation study of the RCAC, [49] were used as primary outcomes for the Fex-Can Fertility trial.<br><br>Secondary outcome measures<br>Health-related quality of life<br>Health-related quality of life was measured with the validated [50] summary score (range 0-100) of the EORTC-QLQ-C30 version 3.0 questionnaire which is a generic instrument for all cancer diagnoses [51].<br><br>Emotional distress<br>The Hospital Anxiety and Depression Scale (HADS) is a widely used scale measuring anxiety (7 items) and depression (7 items), validated for use in cancer patients [52].<br>Scores are given on a numbered Likert scale ranging from 0 through 3 and for each subscale a total score 0-21 (with higher values indicating more anxiety/depressive symptoms) is calculated.<br><br>Fertility self-efficacy<br>Perceived confidence in one's ability to manage situations and emotions related to the threat of infertility was measured with a study-specific questionnaire based on previous research [53, 54], including six items with statements such as: "I feel confident that I can tell other people I'm concerned about my reproductive ability". All the items are available in Supplementary file 1. Answers were given on a 4-point Likert scale with alternatives ranging from completely disagree (1) to agree completely (4). An exploratory factor analysis (data not shown) indicated that one of the items was poorly correlated with the others. A mean score was calculated from the five remaining items, with higher values indicating higher levels fertility-related self-efficacy.<br><br>Fertility-related knowledge<br>The perceived level of knowledge concerning fertility issues was measured using a study-specific questionnaire developed from previous research [18] and consisting of 10 items. Answers were given on a 4-point Likert scale with alternatives ranging from completely disagree (1) to agree completely (4). Exploratory factor analysis (data not shown) on the total cohort of eligible participants indicated it was suitable to divide the scale into two domains; one for general fertility-related knowledge (4 items) and one for cancer-related fertility knowledge (6 items). Items included statements such as: "I have good knowledge regarding the menstrual cycle and when a pregnancy can occur" (general fertility knowledge) and "I have good knowledge regarding the effect of cancer and cancer treatments on reproductive ability" (cancer-related fertility knowledge). All the items are available in Supplementary file 1. Means were calculated for each subscale, with higher mean scores indicating better perceived knowledge"		

**8a) CONSORT: Method used to generate the random allocation sequence**		
"Allocation (1-1 ratio) to either intervention (IG) or control group (CG) was performed by an external statistician uninvolved in the data collection process, by stratified block randomization taking account of sex and diagnosis."		
**8b) CONSORT: Type of randomisation; details of any restriction (such as blocking and block size)**		
"by stratified block randomization taking account of sex and diagnosis."		
**9) CONSORT: Mechanism used to implement the random allocation sequence (such as sequentially numbered containers), describing any steps taken to conceal the sequence until interventions were assigned**		
"Randomization was performed by an external statistician uninvolved in the data collection process"		
**10) CONSORT: Who generated the random allocation sequence, who enrolled participants, and who assigned participants to interventions**		
"Randomization was performed by an external statistician uninvolved in the data collection process". Participants were enrolled using national registries. Invitation letters were sent by menmbers of the research team or an administrator.		
**11a) CONSORT: Blinding - If done, who was blinded after assignment to interventions (for example, participants, care providers, those assessing outcomes) and how**		
**11a-i) Specify who was blinded, and who wasn't**		
"Due to the design of the intervention a placebo condition was not possible and neither participants nor researchers could be blinded to group allocation. "		
**11a-ii) Discuss e.g., whether participants knew which intervention was the "intervention of interest" and which one was the "comparator"**		
**11b) CONSORT: If relevant, description of the similarity of interventions**		
Not relevant since there was only one intervention		
**12a) CONSORT: Statistical methods used to compare groups for primary and secondary outcomes**		
"Data were analyzed using descriptive and inferential statistics. The statistical analyses were performed by external statisticians on blinded data."<br>"T-tests were used to determine any significant differences between the IG and CG at baseline (T0), directly after the 12-week intervention (T1) and 3 months later (T2)."<br>"Linear mixed models were then used to analyze possible change over time within and between treatment groups on the main outcome measure. "		
**12a-i) Imputation techniques to deal with attrition / missing values**		
"Missing data were treated as follows: For single items that were missing, we imputed according with the individual's mean on the scale, provided half or more of the items had been answered. We chose not to impute for individuals where the entire scale was lacking (1-3 participants per group, see Table 3."		
**12b) CONSORT: Methods for additional analyses, such as subgroup analyses and adjusted analyses**		
"Two types of subgroup analyses were then performed. First, for each dimension, participants were assigned to either "high RCAC" (≥4) or "low RCAC" (<4) on the subscale mean at baseline. In the second subgroup analysis participants were stratified based on three levels of adherence to the program (high, low, control)."		
RESULTS		
**13a) CONSORT: For each group, the numbers of participants who were randomly assigned, received intended treatment, and were analysed for the primary outcome**		
"Randomization resulted in 64 persons in the intervention group (IG) and 60 in the control group (CG). "		
**13b) CONSORT: For each group, losses and exclusions after randomisation, together with reasons**		
This is shown in the manuscript in a CONSORT flow diagram		
**13b-i) Attrition diagram**		
. "Participants were considered lost to follow-up only if they for any reason did not return post-intervention questionnaires; hence no pattern of attrition was determined post-randomization. "		
**14a) CONSORT: Dates defining the periods of recruitment and follow-up**		
"The sample was drawn from a cohort of 1499 individuals diagnosed with breast, cervical, ovarian or testicular cancer, lymphoma, or CNS tumor between 2016-2017, approximately 1.5 years prior to the start of the study."		
**14a-i) Indicate if critical "secular events" fell into the study period**		

**14b) CONSORT: Why the trial ended or was stopped (early)**		
Not relevant since the trial did not stop early.		
**15) CONSORT: A table showing baseline demographic and clinical characteristics for each group**		
"Participant characteristics including socio-demographic and clinical variables are summarized in Table 1."		
**15-i) Report demographics associated with digital divide issues**		
A majority had a partner, were working as their main occupation and had a university/college level of education		
**16a) CONSORT: For each group, number of participants (denominator) included in each analysis and whether the analysis was by original assigned groups**		
**16-i) Report multiple "denominators" and provide definitions**		
Number of participants at each measurement point is reported in the tables.		
**16-ii) Primary analysis should be intent-to-treat**		
"All the available data were used, and the analysis was based on the intention-to-treat principle."		
**17a) CONSORT: For each primary and secondary outcome, results for each group, and the estimated effect size and its precision (such as 95% confidence interval)**		
This information is presented in the tables		
**17a-i) Presentation of process outcomes such as metrics of use and intensity of use**		
**17b) CONSORT: For binary outcomes, presentation of both absolute and relative effect sizes is recommended**		
Not relevant since we don't have any binary outcomes.		
**18) CONSORT: Results of any other analyses performed, including subgroup analyses and adjusted analyses, distinguishing pre-specified from exploratory**		
"Including RCAC baseline scores and activity in the program, respectively, did not substantially change the results and did not produce any clear pattern (data available in Supplementary files 4-5)."		
**18-i) Subgroup analysis of comparing only users**		
**19) CONSORT: All important harms or unintended effects in each group**		
"One person (2%) experienced a worsening and commented that this was not due to the program"		
**19-i) Include privacy breaches, technical problems**		
**19-ii) Include qualitative feedback from participants or observations from staff/researchers**		
DISCUSSION		
**20) CONSORT: Trial limitations, addressing sources of potential bias, imprecision, multiplicity of analyses**		
**20-i) Typical limitations in ehealth trials**		
"Randomized controlled trials are usually considered the gold standard for scientific evidence. However, in social and psychological interventions, and eHealth interventions especially, conditions are not fully controlled as double blinding is not possible. The researchers cannot influence what type of accessory support either the intervention or control group have access to, and substantial amounts of self-help information are readily available on websites, via social or traditional media. This may lead to inconclusive assessment of intervention effects. Furthermore, there are various sources of bias introduced by design choices such as not having a set standard for adherence, e.g., homework or a minimum assignment for participants. "		
**21) CONSORT: Generalisability (external validity, applicability) of the trial findings**		
**21-i) Generalizability to other populations**		

**21-ii) Discuss if there were elements in the RCT that would be different in a routine application setting**		
This question is discussed more in detail in another publication (doctoral thesis)<br>"The Fex-Can Fertility program could be useful for improving knowledge about fertility and for reducing concerns about genetic risks following cancer. The automated, flexible, and partially tailorable design of the intervention makes it convenient as a tool in clinical care."<br><br>"Further research about the mechanisms of impact is required to determine for whom the Fex-Can program or similar interventions may constitute an appropriate individualized support. "		
**22) CONSORT: Interpretation consistent with results, balancing benefits and harms, and considering other relevant evidence**		
**22-i) Restate study questions and summarize the answers suggested by the data, starting with primary outcomes and process outcomes (use)**		
"The aim was to determine the capacity of this web-based self-help program in alleviating fertility distress as measured with the RCAC scale. Assessment at follow-up 3 months after the end of the program showed significant differences in one out of six dimensions of the RCAC scale, Child's health, where the intervention group had less distress than the control group. For the secondary outcome cancer-related fertility knowledge, the intervention group reported better knowledge than the control group both directly post-intervention and at the 3-month follow-up. Effect sizes were small to moderate, with a more pronounced effect at the 3-month follow-up. Subgroup analyses assessing the possible interaction effect of time x group, adherence and baseline RCAC scores on the main outcome measure did not substantially alter the results."		
**22-ii) Highlight unanswered new questions, suggest future research**		
Other information		
**23) CONSORT: Registration number and name of trial registry**		
"Trial registration number<br>ISRCTN36621459. Registered 25 January 2016."		
**24) CONSORT: Where the full trial protocol can be accessed, if available**		
The paper contains a reference to the protocol of the RCT.		
**25) CONSORT: Sources of funding and other support (such as supply of drugs), role of funders**		
"Our gratitude goes to all participants of the Fex-Can intervention, and to our funders. "<br>Funders are listed separately from the manuscript.		
**X26-i) Comment on ethics committee approval**		
"The present study was approved by the Regional Board of Ethics in Stockholm (permits no 2013/1746-31/4; 2014/224-32; 2017/916-32) and performed in accordance with the ethical standards as laid down in the 1964 Declaration of Helsinki and its later amendments."		
**x26-ii) Outline informed consent procedures**		
**X26-iii) Safety and security procedures**		
**X27-i) State the relation of the study team towards the system being evaluated**		
Authors declare that they have no conflict of interest		
